# Genome-wide siRNA Screening at Biosafety Level 4 Reveals a Crucial Role for Fibrillarin in Henipavirus Infection

**DOI:** 10.1371/journal.ppat.1005478

**Published:** 2016-03-24

**Authors:** Celine Deffrasnes, Glenn A. Marsh, Chwan Hong Foo, Christina L. Rootes, Cathryn M. Gould, Julian Grusovin, Paul Monaghan, Michael K. Lo, S. Mark Tompkins, Timothy E. Adams, John W. Lowenthal, Kaylene J. Simpson, Cameron R. Stewart, Andrew G. D. Bean, Lin-Fa Wang

**Affiliations:** 1 CSIRO Health and Biosecurity, Australian Animal Health Laboratory, Geelong, Victoria, Australia; 2 Victorian Centre for Functional Genomics, Peter MacCallum Cancer Centre, East Melbourne, Victoria, Australia; 3 CSIRO Manufacturing, Parkville, Victoria, Australia; 4 Centers for Disease Control & Prevention, Viral Special Pathogens Branch, Atlanta, Georgia, United States of America; 5 Department of Infectious Diseases, University of Georgia, Athens, Georgia, United States of America, and School of Medicine, Deakin University, Waurn Ponds, Victoria, Australia; 6 The Sir Peter MacCallum Department of Oncology, The University of Melbourne, Melbourne, Australia; 7 Program in Emerging Infectious Diseases, Duke-NUS Graduate Medical School, Singapore; Icahn School of Medicine at Mount Sinai, UNITED STATES

## Abstract

Hendra and Nipah viruses (genus *Henipavirus*, family *Paramyxoviridae*) are highly pathogenic bat-borne viruses. The need for high biocontainment when studying henipaviruses has hindered the development of therapeutics and knowledge of the viral infection cycle. We have performed a genome-wide siRNA screen at biosafety level 4 that identified 585 human proteins required for henipavirus infection. The host protein with the largest impact was fibrillarin, a nucleolar methyltransferase that was also required by measles, mumps and respiratory syncytial viruses for infection. While not required for cell entry, henipavirus RNA and protein syntheses were greatly impaired in cells lacking fibrillarin, indicating a crucial role in the RNA replication phase of infection. During infection, the Hendra virus matrix protein co-localized with fibrillarin in cell nucleoli, and co-associated as a complex in pulldown studies, while its nuclear import was unaffected in fibrillarin-depleted cells. Mutagenesis studies showed that the methyltransferase activity of fibrillarin was required for henipavirus infection, suggesting that this enzyme could be targeted therapeutically to combat henipavirus infections.

## Author Summary

The henipaviruses Hendra and Nipah are bat-borne paramyxoviruses that are highly pathogenic in humans. The need for high biocontainment when studying Hendra and Nipah virus biology has hindered the development of therapeutics and knowledge of the viral infection cycle. This study describes a genome-wide functional genomics screen of human host genes required for henipavirus infection, to our knowledge the first such study conducted at biosafety level 4. Our study demonstrates that henipavirus infection is critically reliant on fibrillarin, a methyltransferase enzyme residing in the cell nucleolus. Despite henipavirus genome replication occurring in the cytoplasm of infected cells, viral RNA synthesis was greatly impaired in cells lacking fibrillarin. Furthermore during the early stages of infection the Hendra virus matrix protein shuttles to the nucleolus and binds fibrillarin. Collectively these results suggest a hitherto unappreciated role for nucleolar host-virus interactions in the early replication phase of henipavirus infection. Finally, mutating the catalytic activity of fibrillarin inhibits henipavirus infection, suggesting that this enzyme could be targeted therapeutically to combat henipavirus infections.

## Introduction

Viruses from the family *Paramyxoviridae* are highly important pathogens impacting human health, collectively causing hundreds of thousands of deaths globally each year [1]. Respiratory syncytial virus (RSV), human metapneumovirus and parainfluenza viruses cause respiratory disease in infants, young children and the elderly. Furthermore, a marked increase in outbreaks of the highly pathogenic Hendra virus (HeV) has recently been observed (35 of 51 reported outbreaks have occurred since 2011) [2], while annual outbreaks of Nipah virus (NiV) in Bangladesh result in 70–100% mortality [3]. Despite the increasing incidence of infections, there are limited vaccines or therapies to treat infections in humans, and many aspects of paramyxovirus pathogenesis remain poorly understood. Such a major global public health problem calls for the development of novel antiviral strategies.

The HeV and NiV genomes (*henipavirus* genus) consist of six genes encoding nine proteins, and the virus exploits host cellular processes to complete its infection cycle. Both viruses enter cells via the ephrin-B2 and/or ephrin-B3 receptors [4]. The reliance on host gene products for virus infection can be determined at a genome-wide level using high-throughput RNA interference (RNAi) screening strategies. There have been many full or partial-genome RNAi screens of host-virus interactions, including orthomyxoviruses [5,6,7,8,9], retroviruses [10,11,12] and flaviviruses [13,14]. To date there has not been a genome-wide siRNA screen of host genes required for paramyxovirus infection.

Genome-wide siRNAs screening involves the use of robotics and advanced imaging equipment, as such there are technical and practical challenges to performing high-throughput screens at biosafety level (BSL)-4, the highest level of physical containment. Thus, previously published genome-wide screens for BSL-4 viruses have used surrogate viruses, such as pseudotyped particles, and have been performed under BSL-2 conditions [15,16]. These surrogate viruses, which often encode only the fusion glycoproteins of the viruses being studied, might not recapitulate the complex and dynamic interactions of all viral genes and proteins acting concurrently in the host cell during a live virus infection. Screen hits from these studies are also often limited to host factors required for early steps of the virus life cycle.

Recent outbreaks of BSL-4 agents such as Ebola virus highlight the need for advanced technological and research capabilities, such as high-throughput experimentation with live viruses at BSL-4, to develop novel intervention strategies. In this study we have performed a genome-wide SMARTpool siRNA screen of protein-coding genes required for HeV infection. To our knowledge, this is the first such study conducted for a paramyxovirus and for a live BSL-4 pathogen. We demonstrate that the henipaviruses require an overlapping subset of host gene products for infection, and that early steps of replication are critically dependent on the nucleolar methyltransferase fibrillarin.

## Results

### Genome-wide analysis of host genes required for henipavirus infection

To facilitate high-throughput screening, we utilized a fully infectious recombinant HeV expressing a luciferase reporter construct [17]. An siRNA library targeting 18,120 protein-coding genes in arrayed format was transfected into HeLa cells that supported both HeV infection and siRNA transfection. After 72 h of target knockdown, cells were infected with recombinant HeV in a BSL-4 laboratory, with luciferase readings taken 24 h post-infection. Cell viability was measured at the time of infection in parallel plates.

**Fig 1:**
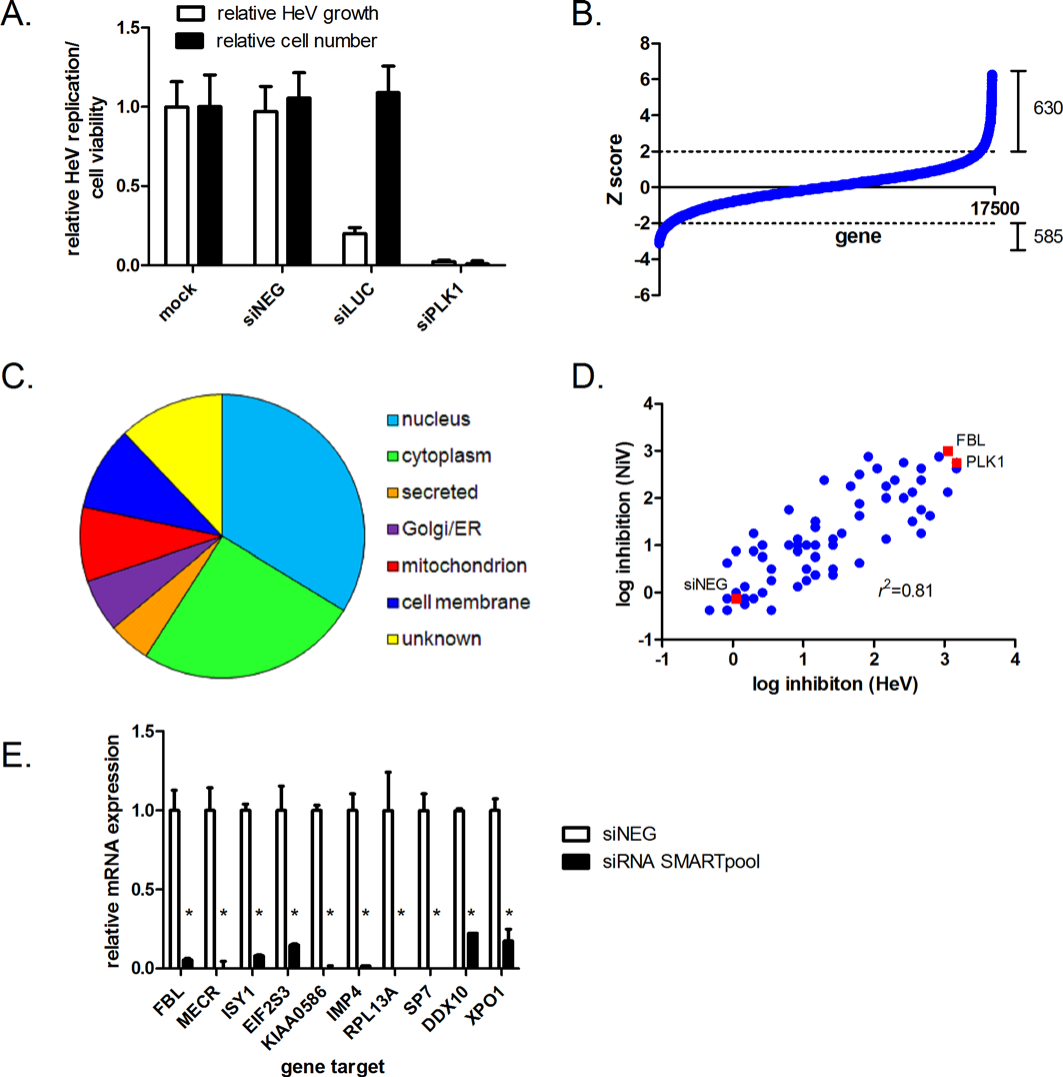
Genome-wide siRNA screen of host genes required for HeV replication. (A) Negative (siNEG), positive (siLUC) and death (siPLK1) control siRNAs used in this study, showing their impact on HeLa cell numbers and growth of recombinant HeV (B) Results from the genome-wide siRNA screen, with genes ranked using a Z score approach, from lowest (decreased virus replication) to highest (increased virus replication). The dotted horizontal lines represent the threshold of statistical significance (Z≥2 or ≤-2). (C) Sub-cellular localization of the 66 high- and medium confidence genes. Genes were manually curated on the basis of subcellular localization annotated in Uni-Prot and GO. (D) Decreases in wild-type HeV and NiV titres during infection (MOI 0.1 for 48 h) caused by silencing of 66 medium- and high-confidence genes. Full details of virus titres are shown in S4 Table. (E) mRNA levels of 10 selected host genes, 72 h post-siRNA transfection. The level of knockdown was normalised to the level of endogenous expression in siNEG transfected cells, set to 1. *p<0.05.

Positive and negative controls were used to evaluate transfection efficiency and assay readout robustness on a per-plate basis. As a negative control, cells were transfected with a SMARTpool siRNA (siNEG) that does not target any gene and that did not impact HeLa cell numbers or HeV infection compared to mock (lipid only transfected) cells (Fig 1A). As a positive control for reducing HeV growth, cells were transfected with an siRNA targeting firefly luciferase (siLUC) that inhibited luciferase reporter expression without impacting cell numbers. Cells were also transfected with a “death control”–a SMARTpool siRNA targeting polo-like kinase 1 (PLK1), a gene associated with apoptosis induction [18]. Death of cells following depletion of PLK1 provided a transfection control in addition to an indirect positive control for inhibition of HeV growth.

Applying a robust Z score normalisation across all screen plates, a typical hit-identification strategy for siRNA screens [19,20], we identified 585 and 630 genes, respectively, that statistically promoted or suppressed HeV infection without adversely impacting cell numbers (Fig 1B, S1 Table and S2 Table, respectively). At the completion of the primary SMARTpool screen, we selected 200 proviral genes based on rank for a secondary screen. Validation was performed by deconvoluting the siRNA pools into the four constituent duplex siRNAs and screening each individually using the same assay format. By this measure, we identified 20 high- and 46 medium-confidence genes (>2 standard deviations from mean mock values for 4/4 or 3/4, or 2/4 siRNAs, respectively) required for HeV infection (S3 Table). We also identified 78 low-confidence (1/4 siRNAs) genes (S3 Table). The sub-cellular localization of the 66 high- and medium-confidence genes is shown in Fig 1C. A noticeable observation is the large proportion of genes, including seven out of the 10 with the greatest impact on virus infection, associated with the nuclear or nucleolar compartments.

We next examined whether the 66 high- and medium-confidence genes required for infection by the reporter HeV were also required by wild-type HeV and the related NiV. Following siRNA-mediated knockdown of candidate genes, HeLa cells were infected with wild-type HeV or NiV (MOI 0.01 for 48 h), and infectious virus titres were measured by TCID_50_ assays. RNAi-mediated silencing of 43 of the 66 genes resulted in significant decreases in HeV titres (S4 Table). The greatest impact was observed from silencing fibrillarin (FBL), which resulted in an approximate 99.9% reduction in virus titres. The majority (41 of 43) of genes required for HeV infection were also required for NiV infection (Fig 1D and S4 Table). Collectively, these data demonstrate that HeV and NiV exploit similar host gene products for infection as well as validating the recombinant virus used in the genome-wide RNAi screen as a faithful reporter of wild-type HeV infection. To verify target knockdown, we measured mRNA levels of 10 host genes, which, according to TCID_50_ analysis, most impacted HeV infection. For all 10 genes, siRNAs targeting these genes caused >80% reduction in gene mRNA expression (Fig 1E).

### FBL is required for henipavirus infection

**Fig 2:**
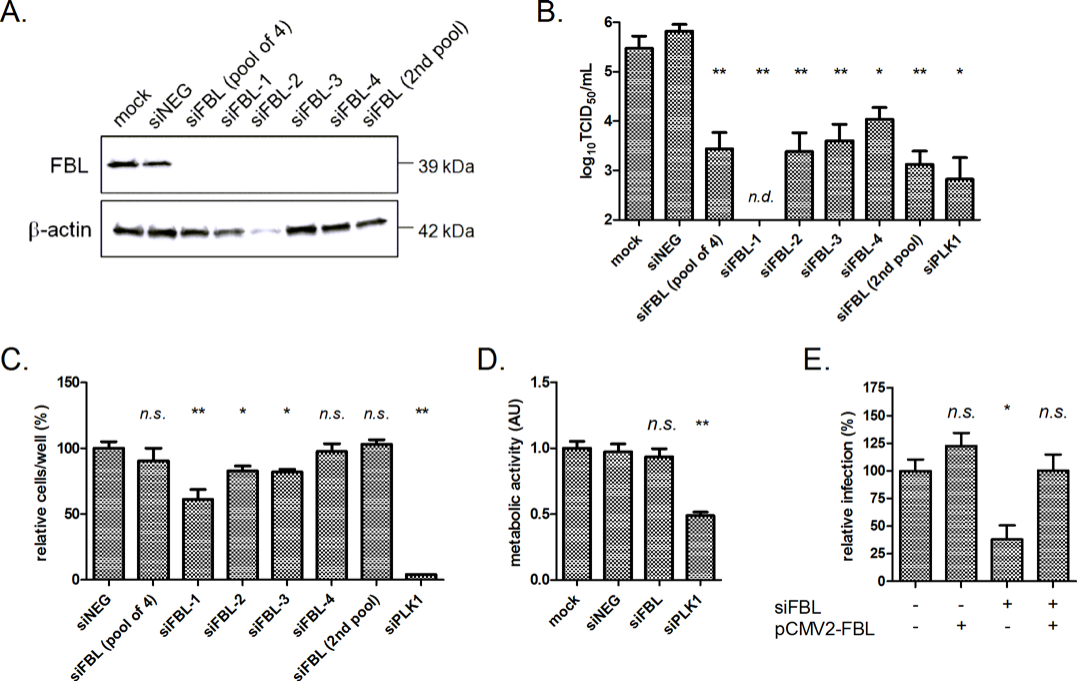
FBL is required for HeV infection. (A) Western blot analysis of HeLa cells transfected with indicated siRNAs. siFBL SMARTpool refers to the 4 siRNA duplexes used to silence FBL in the genome-wide siRNA screen, while siFBL-1, 2, 3, and 4 are the individual constituent duplexes. siFBL (2^nd^ pool) refers to the ON-TARGETplus SMARTpool siRNA with modified chemistry to reduce off-target effects (Dharmacon RNA Technologies). Lysates were collected 72 h post-transfection. (B) HeV titres in HeLa cells infected with HeV (MOI 0.1) for 48 h, 72 h post-transfection with siRNAs. *p<0.05, **p<0.01 compared to siNEG, n.d. not detected. (C) Cell numbers 72 h post-transfection with siRNAs. Data is normalised to siNEG values. *p<0.05, **p<0.01 compared to siNEG, n.s. not significant. (D) Relative cell metabolic activity, measured by Alamar blue assay, in cells transfected with siRNAs as described above. **p<0.01 compared to siNEG, n.s. not significant compared to siNEG. (E) HeV infection in cells depleted of endogenous FBL +/- a vector (pCMV2, 500 ng, 24 h) expressing FBL resistant to siRNA, followed by HeV infection (MOI 0.1, 24 h). Relative infection refers to the percentage of cells stained positive for HeV phosphoprotein. *p<0.05 compared to siFBL(-) pCMV2-FBL(-) cells, n.s. not significant compared to siFBL (-) pCMV2-FBL(-) cells.

The requirement of the nucleolar protein FBL for henipavirus infection was intriguing given that, like most negative-stranded RNA viruses, henipaviruses replicate in the cell cytoplasm [21]. As both HeV and NiV infection was inhibited more than 99.9% by FBL knockdown (S4 Table), we investigated the role of FBL in HeV infection in greater detail. We first validated the knockdown of FBL at the protein level. Cell lysates collected from HeLa cells transfected with siNEG showed a single band corresponding to FBL (~39 kDa) by Western blotting (Fig 2A). In contrast, lysates from cells transfected with the SMARTpool siFBL siRNA used in the genome-wide screen, or the constituent siRNA duplexes, showed no detectable FBL protein at 72 h post transfection. A second ON-TARGETplus SMARTpool of siFBL siRNAs with distinct nucleoside sequences featuring chemical modification to reduce off-target effects [22] also depleted FBL (Fig 2A, siFBL (2^nd^ pool)).

We next assessed the impact of FBL knockdown on the production of infectious wild-type HeV. Using the SMARTpool siFBL, or the individual duplexes that comprised the pool, a significant reduction (>99%) in HeV titers was observed in supernatant collected 48 h after infection (Fig 2B). The ON-TARGETplus SMARTpool also inhibited HeV infection, providing further confidence that HeV infection was inhibited by the loss of FBL.

We next assessed the impact of FBL knockdown on cell health. Transfecting HeLa cells with individual duplexes siFBL-1, -2 and -3 showed a mild impact on cell numbers in conjunction with excellent target knockdown. For siRNA-4 and both SMARTpools, cell numbers were not significantly different to mock or siNEG controls (Fig 2C). An Alamar blue assay showed no significant change in metabolic activity in cells treated with the siFBL SMARTpool for 72 h, compared to cells treated with siNEG (Fig 2D). By contrast, cells transfected with siPLK1 showed significantly reduced metabolic activity.

To unambiguously demonstrate that the reduction in HeV infection was mediated by the absence of FBL we conducted a rescue experiment. HeLa cells were transfected with siFBL-2 to knock down endogenous FBL expression, then transfected with a plasmid encoding a FBL gene containing silent mutations in the siFBL-2 target sequence (pCMV2-FBL) followed by infection with HeV. Transfecting cells with siFBL-2 decreased the percentage of cells with detectable viral antigen staining by ~70%. Co-transfection of siFBL and pCMV2-FBL successfully restored HeV infection to a level similar to non-transfected cells (Fig 2E). Notably, over-expressing FBL with pCMV2-FBL did not increase HeV infection compared to control cells. This was further confirmed by a separate experiment where pCMV2-FBL over-expression was titrated (S1 Fig).

Importantly, silencing FBL using the SMARTpool siRNA depleted FBL protein, inhibited HeV infection but did not negatively impact cell numbers or cell viability. All of the subsequent experiments were therefore performed with siFBL SMARTpool unless indicated.

### FBL is required for infection by diverse paramyxoviruses

**Fig 3:**
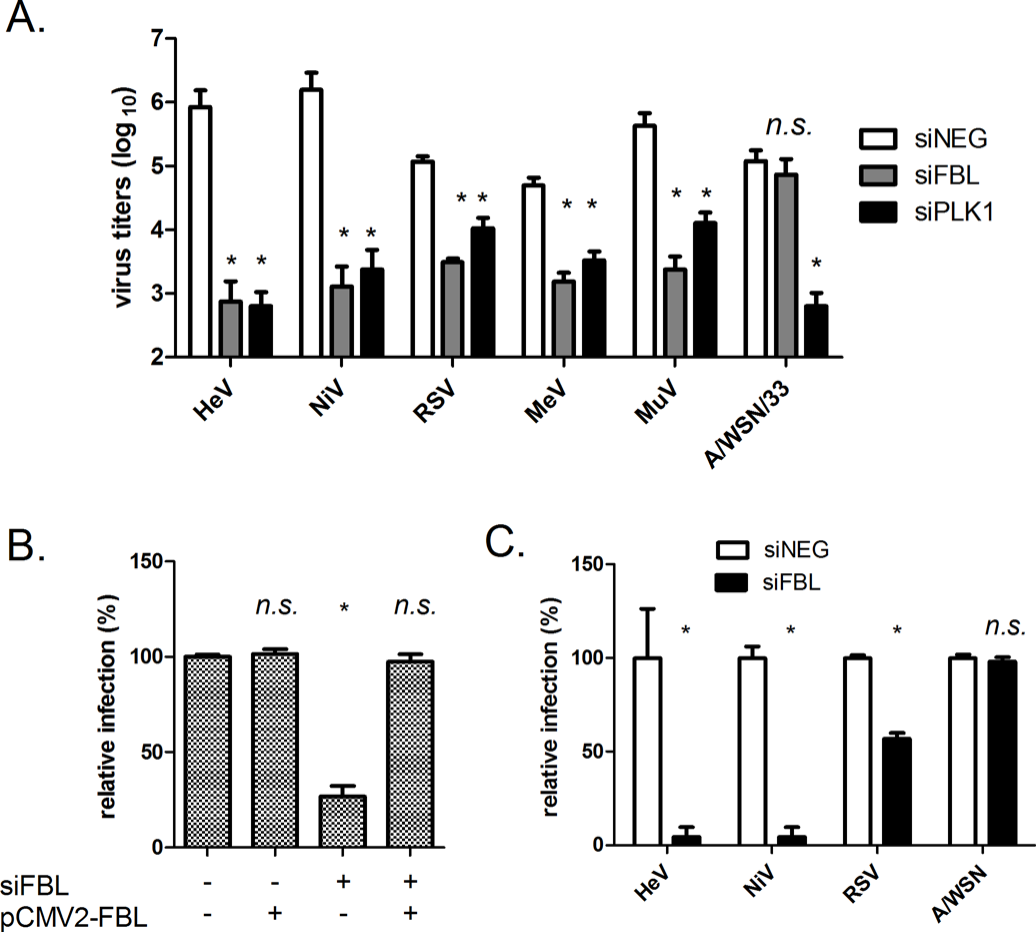
Depletion of FBL inhibits infection by diverse paramyxoviruses. (A) Virus titres in HeLa cells infected with indicated viruses (MOI 0.1) for 48 h, 72 h post-transfection with indicated siRNAs. *p<0.05 compared to siNEG, ns. not significant compared to siNEG. (B) RSV infection in cells depleted of endogenous FBL +/- a vector expressing FBL resistant to siFBL-2, followed by RSV infection (MOI 0.1, 48 h). *p < 0.05 compared to siFBL(-) pCMV2-FBL(-) cells, n.s. not significant compared to siFBL(-) pCMV2-FBL(-) cells. (C) Relative infection of HeLa cells infected with indicated viruses as above, normalised to siNEG. *p<0.05, compared to siNEG, n.s. not significant compared to siNEG.

We sought to determine whether other paramyxovirus infections were dependent on FBL. Members of the family *Paramyxoviridae* are divided into two subfamilies (*Paramyxovirinae* and *Pneumovirinae*) where HeV and NiV belong to the genus *Henipavirus* in the subfamily *Paramyxovirinae*. We opted to test viruses belonging to different genera in the same subfamily: measles virus (MeV, genus *Morbilivirus*) and mumps virus (MuV, genus *Rubulavirus*), and a virus belonging to the subfamily *Pneumovirinae*, RSV (genus *Pneumovirus*). Genome replication for all of these viruses occurs solely in the cytoplasm. In addition, since influenza viruses (family *Orthomyxoviridae*) are known to replicate primarily in the host nucleus [23], a laboratory strain (A/WSN/33), which has been used in a number of RNAi screens [5,6], was included in our study for comparison. We observed a significant reduction in virus titres for HeV, NiV, RSV, MeV and MuV but not for A/WSN/33 in cells transfected with siFBL (Fig 3A). To confirm that the reduction in the other paramyxoviruses was caused by FBL depletion, the rescue experiment described earlier (Fig 2E) was repeated with RSV. Fig 3B demonstrates that FBL is a factor required for RSV infection. We also used immunofluorescence imaging and software quantification as the readout for virus infection. Cells transfected with siNEG, then infected with HeV for 48 h, showed extensive HeV phosphoprotein (P) staining, whereas cells depleted of FBL showed almost no viral protein expression (Fig 3C). Depleting cells of FBL also significantly reduced the proportion of cells stained for RSV nucleoprotein (Fig 3C), however, not to the same extent as HeV or NiV. By contrast, depleting cells of FBL did not alter influenza A/WSN/33 nucleoprotein staining (Fig 3C). FBL is the catalytic component of small nucleolar ribonucleoprotein (snoRNP) complex responsible for site-specific 2’O-methylation of ribose found on ribosomal RNAs [24]. Pre-ribosomes undergo chemical modifications such as 2’O-methylation and pseudouridylation as part of their processing during ribosomal biogenesis [24]. As depleting cells of FBL would reasonably be expected to impact ribosome biogenesis and protein translation, and therefore may inhibit virus infections non-specifically, the lack of inhibition of A/WSN/33 infection in FBL-depleted cells demonstrates that FBL is specifically required for paramyxovirus infection. This is also supported by previous RNAi screens that do not link FBL with the infection cycle of several strains of influenza virus [5,6,7,8,9].

### FBL is essential for the early stages of henipavirus infection post-entry

**Fig 4:**
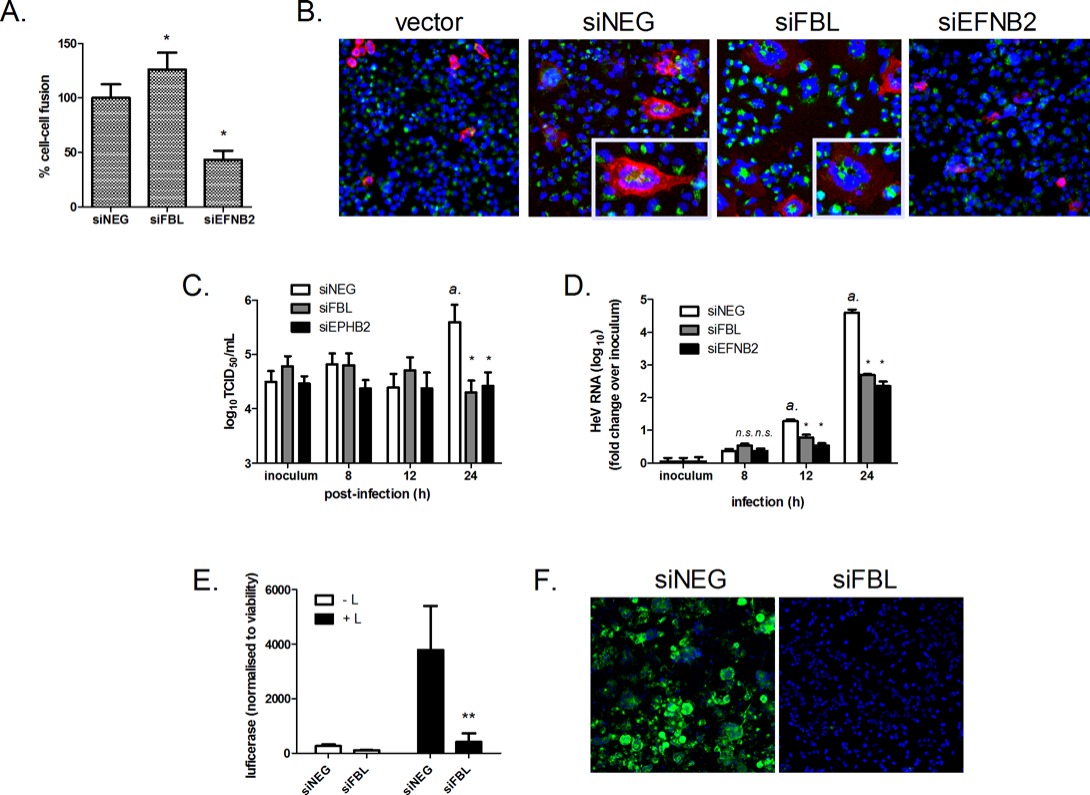
FBL is essential for the early stages of HeV infection post-entry. (A) Cell-to-cell fusion of HeV-F and HeV-G-expressing (effector) HEK-293T cells to (target) HeLa cells treated with indicated siRNAs. Values are normalised to siNEG, set to 100. *p<0.05 compared to siNEG. (B) Typical fusion visualised by immunofluorescence. Nuclei are shown in blue, target cells green and effector cells red. (C) TCID_50_ measurements of virus titres and (D) qRT-PCR measurements of intracellular viral RNA in HeLa cells infected with HeV (MOI 5). *a*: p<0.05 compared to inoculum, *p<0.05 compared to siNEG. HeV RNA values were normalised to cellular 18S levels and to inoculum levels of HeV, set to 1. (E) Levels of transcription and replication in a plasmid-based Nanoluciferase reporter minigenome assay. HeLa cells were transfected with HeV replication support plasmids and minigenome plasmid in cells transfected with siNEG or siFBL. Reporter activity levels were normalised to cell viability (cellular ATP levels). **p<0.01 compared to siNEG. (F) Immunofluorescence microscopy showing HeV-P protein staining (green) in HeLa cells transfected with siNEG or siFBL, followed by HeV infection (MOI 0.1, 24 h).

We sought to determine which stage of the henipavirus infection cycle is dependent on FBL. We first tested the impact of FBL on HeV cell entry using an established cell-cell fusion assay [25]. When incubated with effector cells expressing HeV-F and HeV-G proteins, target cells depleted of FBL exhibited modestly higher fusion levels with effector cells compared to target cells transfected with siNEG (Fig 4A and 4B), suggesting that FBL is not required for HeV cell entry. As a positive control, depleting cells of the HeV entry receptor ephrin-B2 (using a SMARTpool siRNA, siEFNB2) decreased cell-cell fusion by 70% relative to siNEG.

In order to investigate whether FBL is required for viral genomic replication and transcription, we first performed a timecourse experiment to characterize the single-cycle replication kinetics of HeV in HeLa cells. A previous study has shown that in HeLa cells, NiV buds from the plasma membrane at or prior to 24 h post-infection (p.i.), concurrent with the earliest timepoint for observable syncytia [26]. Consistent with this report, HeLa cells infected with a high MOI of HeV started producing infectious virions (above inoculum levels) between 12 and 24 h p.i. (Fig 4C). This indicates that the length of one cycle of HeV infection in HeLa cells is approximately 12 to 24 hours. Consequently, a validated TaqMan qPCR assay [27] was used to measure intracellular viral RNA levels at 8, 12 and 24 h p.i.. Intracellular viral RNA levels started increasing above inoculum levels between 8 and 12 h p.i. (Fig 4D), which is consistent with the replication kinetics observed with the virus production data (Fig 4C). More importantly, within this single-cycle infection period (12 h p.i.), knockdown of either FBL or EFNB2 significantly reduced intracellular viral RNA levels relative to siNEG. Additionally, at 24 h p.i., virus titers in siFBL and siEFNB2 groups showed significantly reduced levels compared to siNEG (Fig 4C).

Finally, the impact of FBL on viral replication was also assessed in a minigenome assay not involving infectious virus. HeV gene expression levels were significantly reduced in HeLa cells transfected with siFBL compared to siNEG (Fig 4E). As expected, viral protein production (P protein and N protein) was almost completely abolished in HeV-infected cells treated with siFBL (Fig 4F and S2 Fig, respectively). Collectively, these results indicate that FBL is required for viral RNA synthesis during the pioneering rounds of infection.

### FBL binds HeV matrix protein (HeV-M) but is not required for HeV-M nuclear localization early during infection

**Fig 5:**
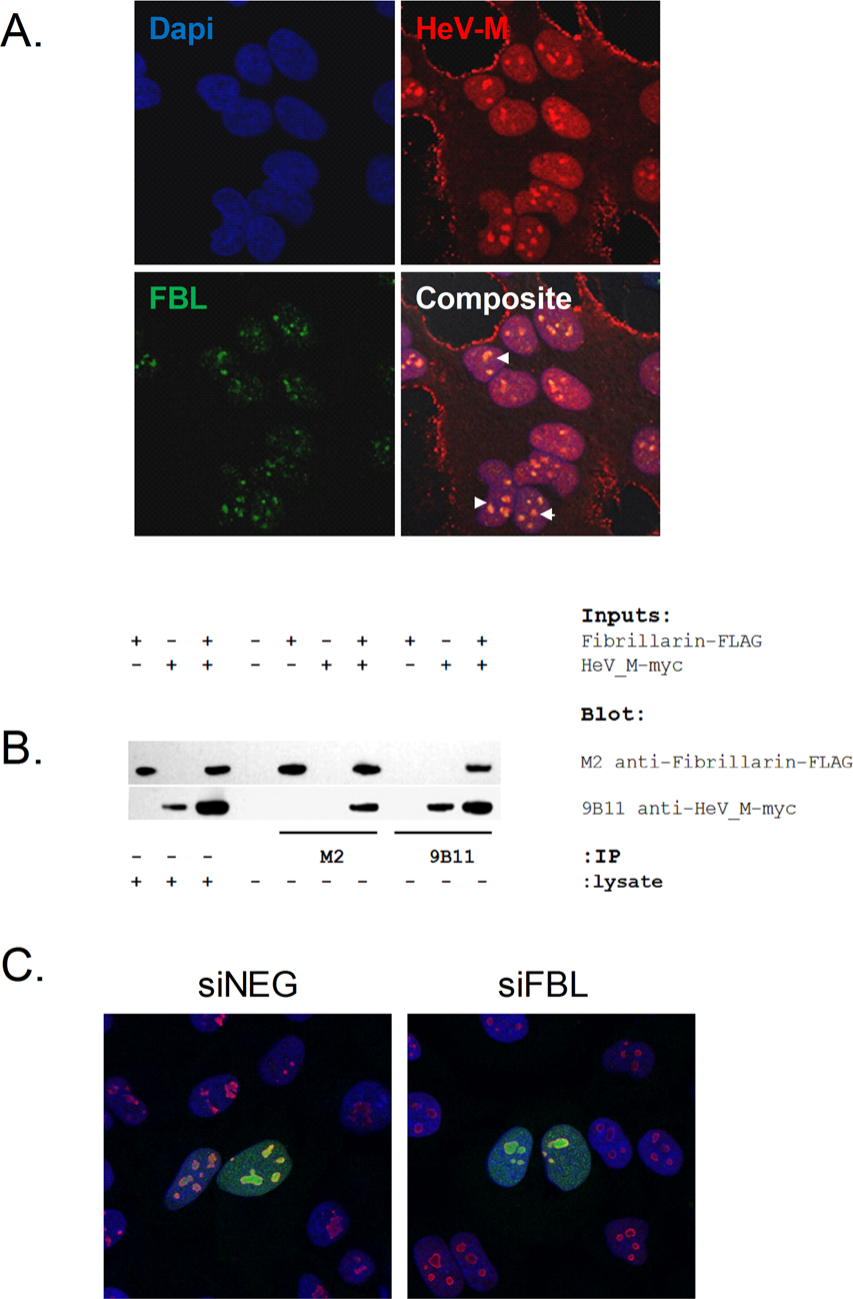
FBL and HeV-M localize to the nucleolus and bind to each other. (A) Confocal microscopy of HeLa cells infected with HeV (MOI 0.1) for 24 h. In the merged image, co-localization of HeV-M (red) and FBL (green) appears as areas of yellow (arrows). (B) HEK293T cells were transfected to express FLAG-tagged FBL and myc-tagged HeV-M proteins either alone or in combination. Lysates were immunoprecipitated with M2 anti-FLAG or 9B11 anti-myc MAbs, separated by 4%-12% Bis-Tris PAGE and transferred to a nitrocellulose membrane for Western blotting. Duplicate blots were probed with either M2-HRP or 9B11-HRP to reveal reciprocal co-immunoprecipitation. (C) Confocal microscopy of HeLa cells transfected with siNEG or siFBL for 72h and then transfected with a myc-tagged HeV-M expressing plasmid for 12h. Nuclei are shown in blue, myc-tagged HeV-M green and nucleolin red.

FBL, along with 18 out of 43 other validated high-confidence hits, localizes primarily to the nucleus or nucleolus of the cell (S3 and S4 Tables). Interestingly, even though henipaviruses replicate in the cytoplasm, HeV matrix protein (HeV-M) has been shown to localize mainly in the nucleus early during infection and then throughout the cytoplasm later in infection in mammalian cells [28]. Wang et al. also demonstrated that NiV-M transits through the nucleolus [26]. We investigated whether FBL and HeV-M colocalize in the nucleolus during live virus infection. Using confocal microscopy, we observed the majority of FBL staining in the nucleolus, with weaker staining in the nucleoplasm. Strong HeV-M staining was also found in the nucleolus and localized at the plasma membrane, with weaker staining in the nucleus and cytoplasm (Fig 5A).

Since confocal microscopy suggests a co-localization of FBL and HeV-M in the nucleolus of infected cells, we tested whether the proteins form complexes by reciprocal co-immunoprecipitation. Fig 5B shows that regardless of whether it is FBL or HeV-M that was immunoprecipitated from co-transfected cells, the other protein was detected as a co-immunoprecipitant. Control immunoprecipitations using lysates from single-DNA transfections that express either epitope-tagged FBL or HeV-M demonstrate the specificity of the pull-down. These co-immunoprecipitations, while demonstrating that FBL and HeV-M are associated, do not reveal whether the interaction is direct or indirect via a complex involving other cellular components.

We next considered the functional relevance of the observed physical association between FBL and HeV-M in the nuclear compartment. The role of henipavirus M in the nucleus is unclear, though Wang et al. showed that its nuclear targeting and chemical modification (by ubiquitination) are required prior to its nuclear export and for virion budding during the later stages of viral morphogenesis [26]. The matrix proteins which are targeted into the nucleus during the early stages of henipavirus infection consist of the initial batches of newly synthesized M, and quite likely, the M derived from the original infecting virions (i.e. pioneer M).

Since FBL is required for viral RNA synthesis during early steps of henipavirus replication, any functional relevance of FBL with M should apply primarily to M synthesized prior to or at this stage of infection. Hence, we assessed whether FBL is required for nuclear targeting (import) of M for this stage of the infection cycle. For these experiments, our strategy was to use cells transfected with a cDNA plasmid encoding the ORF of myc-tagged HeV-M, since viral protein synthesis was extremely limited during HeV infection of FBL-knockdown cells (Fig 4F and S2 Fig). Previous studies and those of our own have also shown that the trafficking kinetics of M from a transfected plasmid recapitulates those of M from live henipavirus infections ([26] and S3 Fig). At 12 h post-transfection with plasmid, which is a time prior to HeV-M nuclear egress in live infection, HeV-M was observed largely in the host cell nucleus and nucleolus in cells transfected with siNEG or siFBL and there was no discernible difference in the nuclear and nucleolar import of M between the two treatment groups (Fig 5C), where M colocalized with the nucleolar marker nucleolin. Moreover, in FBL-depleted cells, HeV-M is able to complete its entire functional life cycle and produce virus-like particles, albeit with a reduction in budding efficiency (S4 Fig). Therefore, FBL associates with HeV-M in a nucleolar complex but is not required for M nuclear translocation early during infection.

### Nucleolar snoRNP protein components are required for henipavirus infection

**Fig 6:**
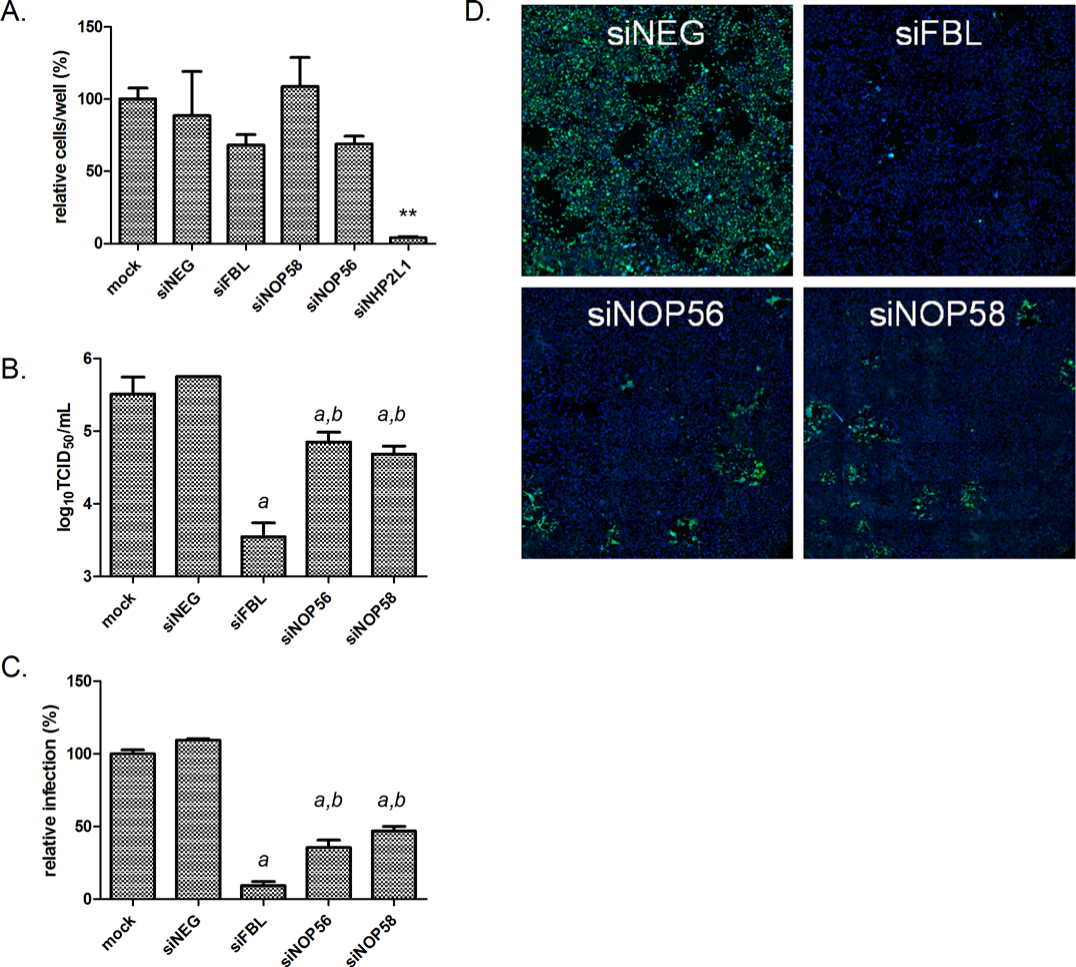
snoRNP protein components are required for HeV infection. (A) Cell numbers 72 h post-transfection with siRNAs. Data is normalised to mock values. **p<0.01 compared to siNEG, (B) TCID_50_ and (C) immunofluorescence measures of virus infection in HeLa cells transfected with siRNAs targeting indicated genes followed by HeV infection (MOI 0.1, 48 h infection). a: *p<0.05 compared to siNEG. b: *p<0.05 compared to siFBL. (D) Immunofluorescence images showing HeLa cells treated as in (A and C) and stained with phosphoprotein-specific (HeV) antibody (green) and DAPI nuclear stain (blue).

In its conventional cellular function of pre-rRNA methylation, human FBL forms a snoRNP complex composed of FBL, NOP56, NOP58, NHP2L1 and small guide RNA molecules [29]. To evaluate whether the reliance of henipaviruses on FBL is mediated through the role of FBL in pre-rRNA methylation, we used SMARTpool siRNAs to reduce expression of every member of the snoRNP complex and measured the impact on HeV infection. Cells depleted of NHP2L1 were not viable, while cells depleted of NOP56 and NOP58 remained viable (Fig 6A). Depletion of either NOP56 or NOP58 inhibited HeV infection, as measured by TCID_50_ assays and immunofluorescence (Fig 6B–6D). However, this impact was significantly less compared to FBL knockdown, which is in agreement with our result from the screen where NOP56 and NOP58 showed an impact on HeV infection but below statistical significance (Z scores -1.99 and -1.05, respectively). These results suggest that the role of FBL in henipavirus infection is mediated via its role in the methylation of pre-ribosomes.

### The catalytic activity of FBL is required for henipavirus infection

**Fig 7:**
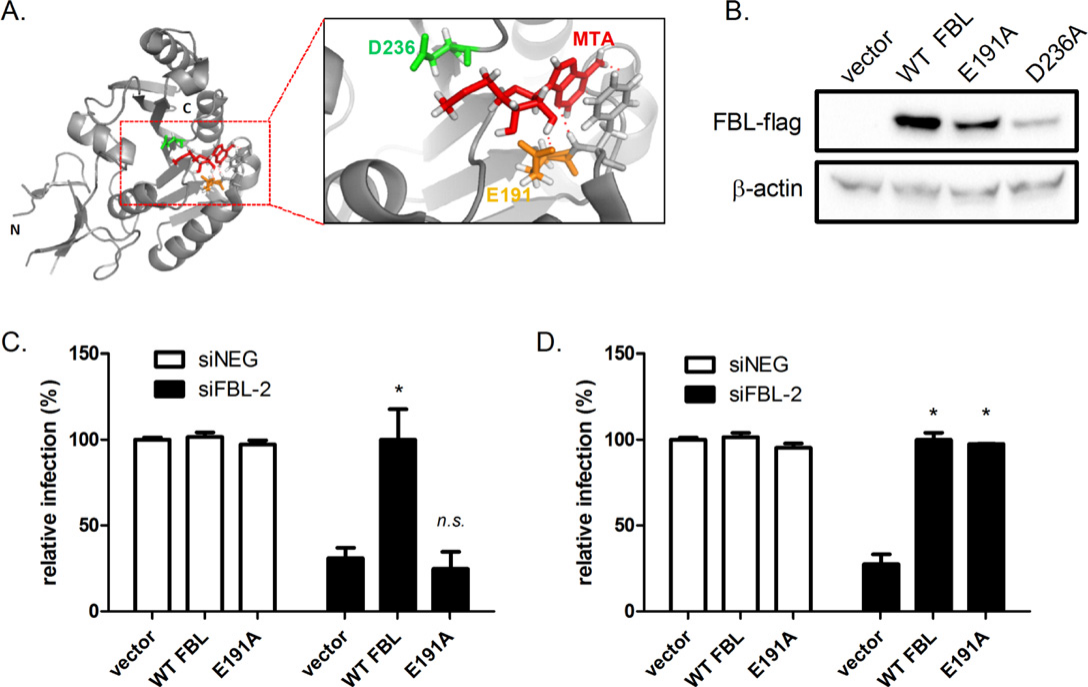
The catalytic activity of FBL is required for HeV infection but not RSV infection. (A) Crystal structure of human FBL (RCSP Protein Databank 2IPX) in complex with 5‘-deoxy-5'-methylthioadenosine (MTA) (red), an analog of the natural cosubstrate factor *S*-adenosylmethionine (SAM). (http://www.rcsb.org/pdb/explore/explore.do?structureId=2IPX). The two conserved residues critical for co-substrate binding are shown. Red lines depict polar contacts between MTA and the huFBL molecule. E191 (orange) and D236 (green) were each substituted with alanine. (B) Protein expression levels of FBL variants assessed by Western blotting of HeLa cell lysates, using an anti-FLAG antibody. HeV infection (C) and RSV infection (D) in HeLa cells depleted of endogenous FBL +/- a vector (pCMV2, 500 ng, 24 h) expressing FBL resistant to siRNA carrying the E191A mutant, followed by HeV infection (MOI 0.1, 24 h). *p<0.05 compared to vector.

FBL is the enzymatic subunit of the snoRNP complex, and acts by catalyzing the transfer of a methyl donor from a bound cofactor *S*-adenosyl methionine (SAM) to riboses of the target pre-rRNA [29]. To assess whether the 2’O-ribose methyltransferase activity of FBL is required for henipavirus infection, we performed a mutagenesis study where the main residues involved in FBL binding to its methyl donor cofactor SAM [30] were mutated. Based on mutagenesis analysis of the yeast FBL (NOP1) [30] and the crystal structure of the human FBL-MTA (a chemical analogue of SAM) complex, two conserved residues critical for FBL catalytic function were selected (Fig 7A). Alanine mutations at yeast residues corresponding to human residues at E191 and D236 result in greatly impaired FBL methylation activity *in vitro* [30]. These mutants were applied to an assay similar to the rescue experiment (Fig 2E), using a cDNA construct expressing recombinant FBL resistant to RNAi silencing. Next, the catalytic residues were individually substituted to alanine residues and expression of the mutants in HeLa cells were confirmed by Western blotting. Expression levels of D236A were low (Fig 7B), possibly due to improper protein-folding, and so this mutant was not tested further. The E191A mutant protein was expressed (Fig 7B) and when tested for its ability to rescue HeV infection, showed impaired virus infection compared to control cells (Fig 7C). On the contrary, the E191A mutant was able to rescue RSV infection (Fig 7D), suggesting that the catalytic activity of FBL is required for henipavirus infection but not for paramyxoviruses from other genera. In addition, co-immunoprecipitation assay indicated that the E191A mutant does not lose binding to HeV-M (S5 Fig). These observations are consistent with the notion that FBL supports henipavirus infection via its role in pre-rRNA processing.

## Discussion

Research into henipaviruses has been limited by multiple factors–not least the fact that few laboratories in the world have appropriate facilities to handle BSL-4 pathogens. Accordingly, many determinants of pathogenesis have remained unclear. Here we present the first high-throughput RNAi screen performed under BSL-4 containment, as well as the findings from a genome-wide analysis of host genes required for live henipavirus infection. The screen resulted in the identification of multiple host genes involved in ribosomal biogenesis (e.g. RPL and RPS family members, ESF1), nuclear export/import (e.g. XPO1, KPNA3) and transcriptional regulation (e.g. BTF3, SP7) that are important for henipavirus infection. Stages of pre-ribosomal processing which are associated with the validated gene hits include rDNA transcription (POLR3E), pre-rRNA cleavage (components of the U3 small subunit processome DDX10 and IMP4) [31], subunit assembly (GTPBP4), chemical modifications of pre-rRNA (FBL, RPL13A) [32,33,34], as well as nuclear export (XPO1) (S3 Table, S6 Fig).

In general, proper ribosomal processing is required for canonical ribosome function (cap-dependent translation) and for efficient cellular and viral protein translation. However, more recent evidence indicates that chemical modifications of pre-ribosomes, (e.g. 2’-O-ribose methylation) are dispensable for downstream canonical ribosome functions [32,33,34]. Undermethylated or non-methylated ribosomes exhibit normal ribosomal RNA processing, polysome formation, global translational activity, and translational fidelity [33]. Interestingly, FBL is the catalytic subunit directly responsible for 2’-O-ribose methylation of pre-rRNA [30], and yet it exhibited the highest impact on henipavirus infection of all the proviral hits from the screen. This suggests that FBL is required by henipaviruses for a unique function other than canonical cap-dependent translation. This notion is consistent with our observations that knockdown of FBL does not affect the synthesis of HeV matrix proteins ectopically expressed from a transfected plasmid (Figs 5 and S4) or the infection and protein synthesis of influenza virus (Fig 3).

Several studies have shown that methylation of rRNA, though not affecting traditional ribosomal functions, does regulate the type of mRNA transcripts targeted for translation [32,33,34]. Specifically, inhibition of rRNA methylation, either by depleting FBL or by drug treatment, inhibits translation of host transcripts with internal ribosome entry site (IRES) elements in their 5’ UTR [33,35]. Interestingly, another validated high-confidence hit from our screen, ribosomal protein L13a (RPL13A) (S4 Table), is also required for rRNA methylation and IRES-mediated translation [33]. Co-immunoprecipitation and colocalization assays further show that FBL associates in a complex with RPL13A and a C/D box snoRNA, U15 [32].

We demonstrate that FBL is not required for henipavirus entry (Fig 4A and 4B) but is instead essential for viral RNA synthesis (Fig 4D and 4E). Consistently, in FBL-deficient cells, the most abundant viral proteins, P and N, were minimally detected (Fig 4F and S2 Fig, respectively). It is conceivable that FBL acts as a proviral factor by modulating the translation of host genes involved in viral replication. Many cellular mRNA transcripts that are IRES-dependent encode for stress response genes [36,37], including inhibitors of apoptosis, cell proliferation factors, and immune-suppressive regulators. The role of FBL in modulating the host microenvironment to support infection will be an interesting subject for future studies.

We have shown that in order to impact henipavirus infection, FBL requires its methylation activity (Fig 7). In eukarya FBL can form a complex with NHP2L1, NOP56 and NOP58 to perform its 2’-O-methyltransferase activity [38] and we have shown that HeV infection is decreased in the absence of any one of the members of this complex (Fig 6), with the exception of NHP2L1 which impacted cell viability. Murray et al reported that disruption of expression of C/D small nucleolar RNAs expression, which also participate in 2’-O-methylation with FBL, confers resistance to viral infection such as RSV [39], thus supporting the idea that host-mediated methylation plays a vital role in paramyxovirus infection. Until recently, FBL was believed to solely modify RNAs, but Tessarz et al demonstrated that FBL is responsible for rDNA transcriptional regulation by methylating Q104 of human histone H2A, opening up the possibility that other host or even viral proteins could be methylated by FBL [40]. In addition to FBL, SET and MYND domain containing 2 polypeptide (SMYD2), also a protein N-methyltransferase, is another validated high-confidence hit (S4 Table). It specifically methylates and dimethylates histone H3, as well as other non-histone proteins [41,42]. This further validates that methylation plays critical roles in henipavirus infection.

We have shown that in cells depleted of FBL and infected with HeV, viral proteins are detected at extremely low levels (Fig 4F and S2 Fig). However, in cells expressing FBL we found that FBL and HeV matrix protein (M) colocalized in the nucleolus (Fig 5A). *Paramyxovirinae* M are known to traffic through the nucleus and nucleolus and that this step is required for efficient viral budding and is regulated by ubiquitination [26,43]. Although current microscopy techniques do not allow detection of M released from the incoming virions (pioneer M), we observed that cells lacking FBL do not show changes in nuclear import of ectopically expressed HeV-M at early timepoints, which suggests that pioneer M can still enter the nucleus even in the absence of FBL (Fig 5C). Moreover, we confirmed binding of FBL and HeV-M (Fig 5B) and although Pentecost et al did not identify FBL as a direct binding partner for *Paramyxovirinae* M, their protein interactome analyses suggested interactions between M and numerous nucleolar proteins such as NOP58 [26,43] which forms a complex with FBL, supporting the idea of a functional interaction between FBL and HeV-M. Moreover, Sun et al demonstrated that the binding between henipaviruses M and nuclear proteins can be disrupted by RNase A treatment, suggesting an indirect binding through cellular RNAs [44]. Interestingly, 19 out of 43 top validated hits have a nuclear or nucleolar localization, emphasizing the importance of nuclear proteins in HeV infection. It has recently been shown that HeV-M and NiV-M bind to the nuclear protein acidic leucine-rich nuclear phosphoprotein 32 family member B (ANP32B), which is involved in nuclear mRNA export processes, regulation of gene expression and apoptosis, and that this binding increases nuclear retention of M [45]. Our screen demonstrated that RNAi-mediated silencing of ANP32B increases (but not significantly) virus infection (Z score 1.54), suggesting that ANP32B may have a hitherto undescribed antiviral role.

*Paramyxovirinae* M main role in viral infection is viral budding and we have shown that viral budding still occurs even in the absence of FBL even though its efficiency was decreased, which suggests that at later times in HeV infection FBL could play a role in M trafficking and viral budding (S4 Fig).

Matrix proteins have also been shown to be involved in regulation of viral transcription [43,46,47], RNA binding [47] and modulation of host transcription [48]. It could be speculated that HeV-M traffics to the nucleolus and interacts with FBL to regulate host gene expression to facilitate viral replication. Alternatively, HeV-M could impact FBL activity such as enhancing its methylation activity.

Future work will aim to understand whether FBL facilitates the infection cycle of paramyxoviruses differentially by more than one mechanism. In this regard, several distinctions between viruses are evident from our study and others: (i) the impact of FBL on virus titres and/or virus protein production were greater for the henipaviruses than MeV, MuV and RSV (Fig 3); (ii) the catalytic activity of FBL was required for HeV infection but not RSV infection (Fig 7), and (iii) while HeV-M traffics to the nucleolus and associates with FBL (Fig 5), RSV-M does not traffic to the nucleolus [48], despite RSV infection being impacted by FBL (Fig 3).

In summary, this screen dataset furthers the understanding of the infection cycle of henipaviruses and is a resource for the study of related paramyxoviruses–a virus family that impacts both human and animal health. This study reveals a previously unappreciated role for nucleolar proteins with methyltransferase activity such as FBL in henipavirus infection, and suggests that methyltransferase enzymes represent a potential target for development of an anti-henipavirus drug. This work also serves as a blueprint for how high throughput RNAi screens can be performed under high biocontainment conditions.

## Materials and Methods

### Cells

HeLa cells (*ATCC* CCL-2), Hep-2 cells (*ATCC* CCL-23), African green monkey kidney epithelial Vero cells (*ATCC* CRL-81), Madin–Darby Canine Kidney (MDCK) cells (*ATCC* CCL-34) and HEK 293T cells (*ATCC* CRL-3216) were maintained as described previously [49,50].

### Viruses

All virology work was conducted at the CSIRO Australian Animal Health Laboratory. Recombinant HeV, wild type HeV (both Hendra virus/horse/1994/Hendra), NiV (Nipah virus/Malaysia/human/99), MeV (wild type Edmonston strain), MuV (Enders strain) and RSV (strain A2) were passaged in Vero cells. Influenza A/WSN/33 (H1N1) (kind gift, Professor Lorena Brown, University of Melbourne) was passaged in the allantoic fluid of 10-day embryonated specific pathogen-free chicken eggs (Australian SPF Services, Cadello, VIC, Australia). All viruses were aliquoted and stored at −80°C for inoculations.

### High-throughput siRNA screening

HeLa cells (1100 cells/well) were transfected in 384-well plates (Corning product # 353988) with siGENOME SMARTpool siRNAs (final concentration 40 nM) using DharmaFECT (DF) 1 lipid transfection reagent (0.03 μL/well) (all reagents from Dharmacon RNAi Technologies, GE, USA). The siCONTROL Nontargeting siRNA #1 (catalog # D-001210-01-05, referred to here as “siNEG”) and a siRNA targeting firefly luciferase (#P-002099-01-20) acted as negative and positive controls respectively. Mock (transfection lipid only) wells were also included. Genome-wide siRNA libraries (catalog numbers in S1 and S2 Tables) were screened at the Victorian Centre for Functional Genomics (VCFG). Each well in the siGENOME SMARTpool siRNA library contained 4 distinct siRNAs targeting different sequences of the target transcript. Deconvolution validation screens were performed using individual siRNA duplexes, all 4 on the same library plate (catalog numbers in S3 Table) at 25 nM with assay conditions as above. Cells were reverse transfected using the Sciclone ALH3000 (Caliper Life Sciences, Hopkinton, MA) and BioTek 406 (BioTek, Winooski, VT) liquid handling robotics. Cells were transfected in quadruplicate and divided into 2 groups of duplicate plates for nuclei quantitation and duplicate plates for HeV infection. At 72 hours post transfection, in parallel with the point of HeV infection, cell viability for each well was assessed by fixing (4% paraformaldehyde for 10 min) and staining plates with the nuclear stain 4',6-Diamidino-2-Phenylindole, Dihydrochloride (DAPI) (Invitrogen, Carlsbad, CA; 1 μg/ml for 20 min in PBS). The number of cell nuclei per well was quantitated for 25 fields using a 20 × objective on a Cellomics Arrayscan VTi microscope using the Target Activation bioapplication of the Cellomics Scan software (iDev workflow). (Thermo Fisher, Waltham, MA). HeV growth was measured in separate plates suitable for luminescence assays (Corning product # 353988). 72 h post-transfection, cells were infected with recombinant HeV (MOI 0.1 using a BioTek 406 liquid handler housed in a class II biosafety cabinet at BSL-4. At 24 hours post-infection, media was removed and 20 μL of PBS added per well. Luminescence was then measured by addition of 20 μL of Bright-Glo Luciferase reagent (Promega, Madison, WI) and reading on a Synergy H4 multimode microplate reader (BioTek).

### Bioinformatic analysis of screen data

Data analysis was automated using a custom R script which combined and analysed the luciferase (HeV growth) and DAPI (cell viability) raw data files to generate a summarized report spreadsheet with raw and normalised values. Both HeV infection and cell viability were normalized to the average of the mock control readout per plate. siRNA transfections that resulted in >50% average reduction in cell viability compared to mock controls were scored as toxic (LC, low cell count) and excluded from further analyses. The experimental robustness was evaluated for each screened plate using the Z’ factor calculation [19], comparing the negative control (siNEG), positive control (siLUC) and death control (siPLK1) for both cell viability and HeV infection. Robust z-scores utilising the median and median absolute deviation (MAD) of all control-normalised sample values were generated across all sample wells and averaged per duplicate plate pair. Robust z score = (sample value-sample median)/sample median absolute deviation were used as the bio-identification method [19,51].

### siRNA transfection

For experiments following the genome-wide screen, HeLa cells (9000 cells/well) were reverse transfected in 96 well plates with siFBL, siNEG or siEFNB2 siGENOME SMARTpool siRNAs (final concentration 40 nM) using DF1 (0.12 μL/well) for 72 h.

### Quantitative real-time PCR

Target gene knockdown was assessed 48 h post transfection with siRNAs using DF1 according to manufacturer’s instructions. RNA purification, cDNA synthesis and quantitative real-time PCR were performed as described previously [49]. HeV RNA was quantified using a HeV Taqman one-step PCR assay described previously [27].

### Western blotting

Protein lysates were collected from cells seeded in 24 well plates, 48 h post-transfection with siRNAs or 24 h post-transfection with plasmid DNA (500 ng/well) using Lipofectamine 2000. Western blot analyses were carried out according to standard protocols with primary antibodies (1:1000) against FBL (monoclonal (38F3), Abcam, Cambridge, UK), β–actin (monoclonal (AC-74), Sigma) or anti-FLAG (CSIRO Manufacturing Flagship) and species-appropriate horseradish peroxidase-conjugated secondary antibody (1:10,000 dilution). Western blot band intensities were quantified using Image Lab software (Bio-Rad).

### TCID_50_ analysis

Assays were performed as described previously [49]. Samples were titrated in triplicate in 96-well plates, co-cultured with Vero cells for three days (HeV, NiV) or seven days (MeV, MuV, A/WSN/33), and assayed for cytopathic effect. The infectious titre was calculated by the method of Reed and Muench [52].

### Plaque assay

Plaque assays were performed using a modified protocol [50]. Briefly, Hep-2 cells were seeded in 24-well plates at 1.9 × 10^5^ cells per well. 50 μL of cell supernatants were added to 150 μL of DMEM, and incubated with HEp-2 cells for 4 h, with shaking every 30 min. Inoculum was removed and replaced by overlay (DMEM/0.3% agarose/2% FCS). Cells were incubated for 7 days at 37°C 5% CO_2_. On day 7, 2 mL of 1% formaldehyde made up in 150 mM NaCl was added, and left to penetrate overnight. Agarose was removed, and 2.5 mL of 0.05% neutral red was added. Wells were stained for 1 h, washed and plaques counted.

### Cell numbers

Viable nuclei were quantified using the CellInsight Personal Cell Imager (Thermo Scientific) at a magnification of 10 ×, with images captured of all areas of each well, as described [49].

### Alamar blue assay

Alamar blue (Invitrogen) assays were performed following manufacturer’s guidelines. Briefly, cells in a 96 well plate were incubated with 10% (v/v) Alamar blue for 4 h at 37°C, followed by fluorescence measured at 570 nm/585 nm excitation emission filter using a Synergy microplate reader (BioTek).

### Immunofluorescence

Cells were stained to detect HeV/NiV-P, HeV-N, HeV-M or NiV-P as described [28]. Cells were stained to detect RSV nucleoprotein (N; mouse monoclonal (8B10), Sapphire Biosciences, used at 1/1000), influenza virus nucleoprotein (NP; mouse monoclonal (AA5H), AbD Serotec, 1/500), FBL (rabbit polyclonal (Ab5821), Abcam, 1/2000), c-myc (mouse monoclonal (clone 9E10) CSIRO Manufacturing Flagship, 1ug/ml) or nucleolin (rabbit polyclonal (Ab22758), Abcam, 1/1000). Cells were stained with 1/200 dilution of the appropriate secondary antibody purchased from Invitrogen (HeV-M, influenza N, RSV N, c-myc: anti-mouse AF488, HeV/NiV-P, HeV-N, FBL: anti-rabbit AF488, FBL, Nucleolin: anti-rabbit AF568). Nuclei were counter-stained with DAPI. Confocal images were acquired using a Leica-microsystems TCS SP5 microscope.

### Quantification of relative antigen staining

HeLa cells in 96 well plates were imaged using the CellInsight at a magnification of 10 x, 49 fields/well representing the entire well. The percentage of infected cells was quantified using the Target Activation bioapplication of the Cellomics Scan software and was determined by dividing the number of antigen-positive cells by the total cell number, multiplied by 100.

### Cell fusion assays

Fusion between F and G-expressing effector cells and permissive target cells was measured using an assay adapted from [25]. Plasmids encoding HeV-F and HeV-G or vector only control were transfected into HEK 293T (effector) cells seeded in 6-well plates (2.5 μg DNA/well, Lipofectamine 2000) and allowed to express for 10 h. HeLa (target) cells were seeded in 96 well plates and transfected with siRNAs (40 nM) for 72 h (as above) and incubated with the Vibrant DiO cell labelling system for 10 min (Life Technologies) to label target cells. Effector and target cell mixtures were incubated in 96-well plates at 37°C for 24 h. The ratio of effector to target cells was 32:1. Cells were fixed with 4% PFA for 30 min, washed with PBS, and stained to detect HeV-G (primary antibody 1:1000 anti-G mouse #185 (21B6), secondary antibody (1:250 anti-mouse Alexa Fluor 568). Nuclei were labeled with DAPI as described above. Cell fusion was measured using the Target Activation bioapplication of the Cellomics Scan software (iDev workflow) on the CellInsight.

### HeV infection timecourse

HeLa cells seeded in 24 well plates (50,000 cells/well) were transfected with siRNAs (final concentration 40 nM) using DF1 as described above. 72 h post-transfection, cells were infected with HeV (MOI 5) for 45 min. After this time, cell media was replaced and following 0 h (inoculum) 6, 8, 12 and 24 h of infection, cell media was collected to measure virion production by TCID_50_ assay and cell lysates collected to detect intracellular viral RNA levels by qRT-PCR.

### Minigenome assay

A Nanoluciferase-based HeV minigenome assay was adapted from a previously developed NiV minigenome assay [53]. Briefly, a bacteriophage T7 polymerase-based HeV minigenome was synthesized (Genscript) expressing a reporter fusion construct of Nanoluciferase (Promega) and mNeonGreen fluorescent protein [54]. The open reading frame encoding the reporter fusion protein was flanked by a T7 promoter, hammerhead ribozyme, and HeV leader and N gene at the 3’ end. HeLa cells (5× 10^3^ per/well were seeded in 96-well plates. The next day, cells were transfected with siRNAs as described above. 48 h post-transfection of siRNAs, appropriate volumes of HeV support plasmids (N (50 ng/well), P (32 ng) and L (50 ng)), HeV minigenome (120 ng) and T7pol (80 ng) were prepared in RNase-free TE buffer. For negative controls, the L plasmid was substituted with an equivalent amount of pcDNA 3.1 plasmid expressing the red fluorescent protein mCherry (Clontech). Plasmids were mixed with 0.6 μL/well LT-1 transfection reagent (Mirus Bio, Madison, WI) and 10 μL Opti-MEM/well. Complexes were incubated for 30 min at room temperature before adding to cells. 48 h post-transfection of minigenome plasmids, 50 μL of Nanoluciferase assay buffer solution (Promega) was added directly to each well. Well contents were transferred to white plates, and after three minutes, luminescence was read on a plate reader (HT-Synergy, Biotek). Cell Titer Glo 2.0 reagent (Promega) was added immediately following reporter minigenome luminescence reading to measure cell viability according to manufacturer’s guidelines. The average raw luminescence value for siNEG transfected cells (n = 16) was set as 100% cell viability. Percent (%) cell viability for individual wells were calculated by dividing their respective raw luminescence values by the average raw luminescence value for siNEG transfected cells. Reporter nanoluciferase activity for each well was then normalised by dividing the nanoluciferase luminescence value by its respective % cell viability as determined above.

### Immunoprecipitation

HEK293T cells cultured in 6-well plates were transfected with 1 μg of pCMV:FLAG-tagged FBL or pCAGGS:myc-tagged HeV-M or both using Fugene6 transfection reagent (Promega). Cells were harvested 48 h post transfection, washed once with 0.5 mL ice-cold PBS and lysed using 450 μL SU buffer (150 mM NaCl, 10% (v/v) glycerol, 1% (v/v) Triton X-100, 1 mM EGTA, 1.5 mM MgCl_2_, 20 mM HEPES pH 7.5), supplemented with protease inhibitors (Roche) and 10 mM EDTA for 20 min on ice. The lysates were centrifuged at 16000 × *g* for 40 min at 4°C and the SU-soluble fractions were recovered. 180 μL aliquots of rec-Protein-A Sepharose 4B (Zymed) were treated with 3 μL of either M2 anti-FLAG Mab (4.2 μg) or 3 μL 9B11 anti-myc Mab (Cell Signaling), incubated at 4°C for 1 h and washed once with 0.5 mL SU buffer to eliminate unbound Mabs. Aliquots of antibody-bound slurry equivalent to 30 μL of original Protein-A Sepharose were resuspended with 200 μL aliquots of lysate for immunoprecipitation for 2 h at 4°C. The slurry was washed three times with SU buffer and resuspended in 1 × reducing PAGE buffer supplemented with 3 M urea.

### Generation of FBL plasmid constructs

The entire ORF of human FBL was amplified by PCR from a cDNA library and cloned into the expression vector pCMV2, generating the pCMV2-FBL plasmid. Site-directed mutagenesis was then performed on pCMV2-FBL using QuikChange Lightning Mutagenesis kit (Agilent Technologies). The resultant plasmid, pCMV2-FBLsi2, has five silent mutations in the target region of a siFBL-2. The forward and reverse primers used for generating pCMV2-FBLsi2 were 5’–gttggtcctcttcttggccagattgattaagtcacggccagagcggtggg– 3’ and 5’–cccaccgctctggccgtgacttaatcaatctggccaagaagaggaccaac– 3’, respectively. Using pCMV2-FBLsi2 as the template, the codons for E191 and D236 critical for catalytic activity were substituted with codons for alanine. Primers used for PCR-based mutagenesis of pCMV2-FBLsi2 were: E191A-F: tagtctatgcagtcgcgttctcccaccgctc, R: gagcggtgggagaacgcgactgcatagacta, D236A-F: gatgtgatctttgctgctgtggcccagccagac, R: gtctggctgggccacagcagcaaagatcacatc. Final sequences of FBL for all plasmids made were confirmed by DNA sequencing.

### FBL mutagenesis assay

HeLa cells seeded in 96 well plates were transfected with either siNEG, siFBL-2, or siFBL SMARTpool. At 24 h post siRNA transfection, the cells were transfected with either 125 ng of pCMV2-FBLsi2, pCMV2-FBLsi2-T173A, pCMV2-FBLsi2-E191A, pCMV2-FBLsi2-F192A, pCMV2-FBLsi2-D236A, or vector only. At 48 h post DNA transfection, the cells were infected with HeV or RSV (MOI 0.1). Inoculum was removed after 1 h, and infection was allowed to proceed for 48 h. The percentage of infected cells was quantified as described above.

### Statistics

The statistical analysis of the screen itself is described above. For all other work, the difference between two groups was analyzed by a two-tailed Student's *t* test and between multiple groups by one-way ANOVA. A *P* value of <0.05 was considered significant. All data points are the average of triplicates, with error bars representing standard deviations. All data are representative of results from at least 2 separate experiments.

## Supporting Information

S1 Table Host genes required for Hendra virus infection. (XLSX)

S2 Table Host genes that inhibit Hendra virus infection. (XLSX)

S3 Table Validation of genes required for Hendra virus infection. (XLSX)

S4 Table Host genes required for infection by wild-type Hendra and Nipah viruses. (XLSX)

S1 Fig Over-expression of FBL does not affect HeV infection. Increasing concentrations of plasmid DNA encoding for human FBL (pCMV2-FBL) were transfected into HeLa cells using Lipofectamine 2000. 48 hrs after transfection, cells were infected with HeV (MOI 0.1). At 48 h.p.i., cell supernatant were harvested and TCID_50_ virus titer analysis were performed on Vero cells. All datapoints were not statistically significantly different (p>0.05, 1-way ANOVA).
(JPG)

S2 Fig Immunofluorescence microscopy showing HeV-N protein staining (green) in HeLa cells transfected with siNEG or siFBL, followed by HeV infection (MOI 0.1, 24 h). (JPG)

S3 Fig Trafficking kinetics of M from a transfected plasmid recapitulates those of M from live henipavirus infection. Immunofluorescence microscopy of HeLa cells transfected with a myc-tagged HeV-M expressing plasmid for 12 h (left) or 24 h (right). Nuclei are stained blue, myc-tagged HeV-M green. Sub-cellular localization of HeV-M at 12 h is primarily nuclear, and cytoplasmic at 24 h.
(JPG)

S4 Fig FBL is required for HeV VLP production. (A) 293T cells undergoing siRNA-mediated knockdown of endogenous FBL were transfected with pCAGGS vector or pCAGGS expressing myc-tagged HeV matrix protein. 48 h post-transfection, whole cell lysates were harvested. Protein expression levels of FBL or M-myc were assessed by Western blotting, using an anti-FBL and an anti-myc antibody, respectively. Detection with an antibody to β-actin were also done as loading controls. (B) Supernatants were harvested from the transfected 293T cells from (A), clarified by low speed centrifugation, and then layered on a 20% sucrose cushion. VLPs were purified by centrifugation at 200,000 ×g for 2 h. VLPs were detected by Western blotting using an anti-myc antibody. Quantification of VLP levels are shown numerically.
(TIF)

S5 Fig Fibrillarin E191A mutation does not abolish M protein interaction. HEK293T cells were transfected to express either FLAG-tagged wild-type or E191A mutant FBL either alone or in combination with myc-tagged HeV-M protein. Lysates were immunoprecipitated with M2 anti-FLAG MAbs, separated by 4%-12% Bis-Tris PAGE and western transferred. Duplicate blots were probed with either M2-HRP or 9B11-HRP to reveal coimmunoprecipitation of HeV-M by FBL.
(TIF)

S6 Fig Functional partnerships and interactions between a subset of the high-confidence validated proviral candidates. The STRING algorithmic database (Search Tool for the Retrieval of Interacting Genes/Proteins) was used to generate a network view of protein-protein interactions, including direct (physical) as well as indirect (functional) associations (http://string-db.org/) (Szklarczyk et al. 2015). The top 43 candidate hits (as validated by TCID_50_ virus titration assay; *p<0.05) were used as input data for the database search. Candidates which are not part of the generated network output are not depicted. The thickness of the lines correlate with the confidence scores of the interactions.
(TIF)
