# Supplementary figures and images for: Genome-wide siRNA Screening at Biosafety Level 4 Reveals a Crucial Role for Fibrillarin in Henipavirus Infection
Source: PLoS Pathog. 2016 Mar 24;12(3):e1005478. doi: 10.1371/journal.ppat.1005478 (PMC4806981; doi:10.1371/journal.ppat.1005478)

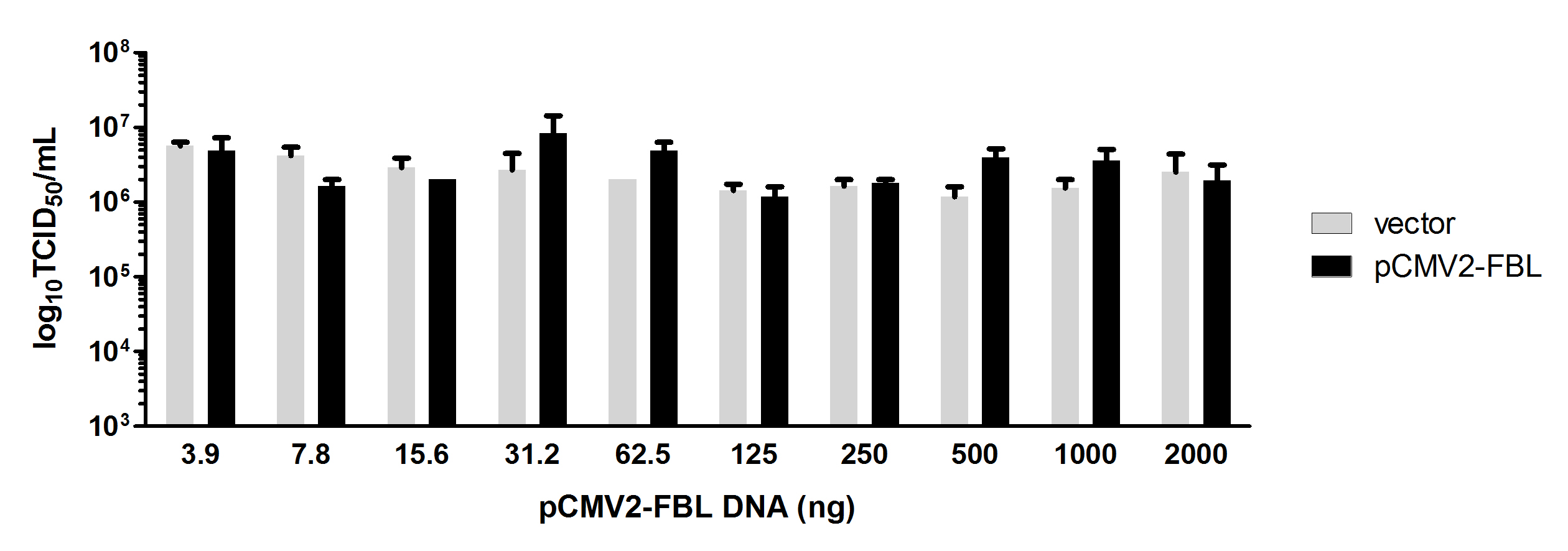

Supplement: S1 Fig — Increasing concentrations of plasmid DNA encoding for human FBL (pCMV2-FBL) were transfected into HeLa cells using Lipofectamine 2000. 48 hrs after transfection, cells were infected with HeV (MOI 0.1). At 48 h.p.i., cell supernatant were harvested and TCID50 virus titer analysis were performed on Vero cells. All datapoints were not statistically significantly different (p>0.05, 1-way ANOVA). (JPG) [file ppat.1005478.s005.jpg]

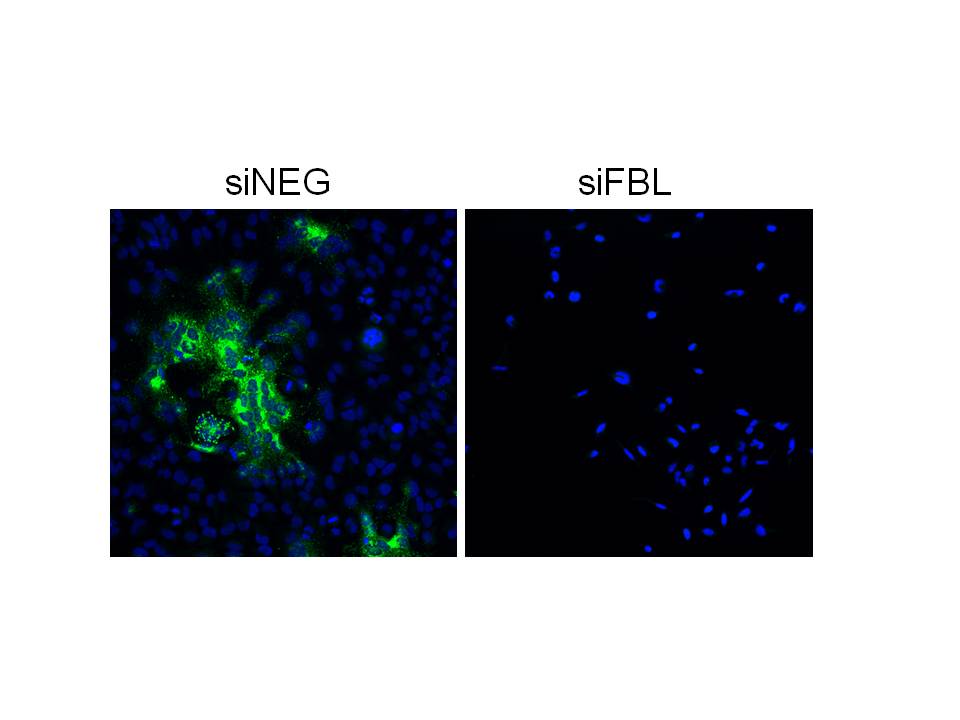

Supplement: S2 Fig — (JPG) [file ppat.1005478.s006.jpg]

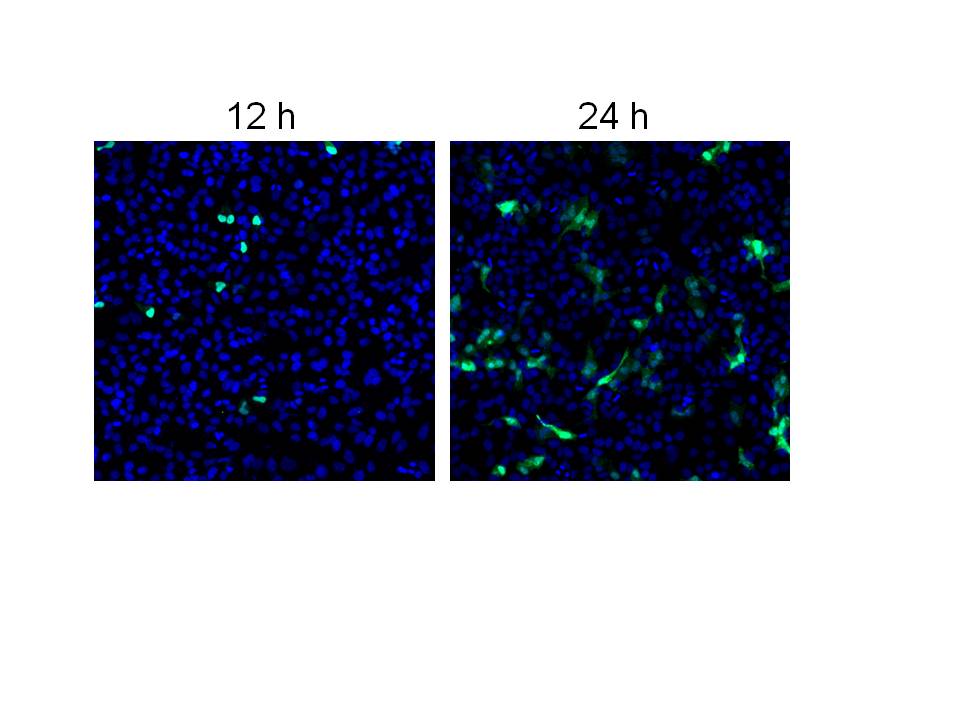

Supplement: S3 Fig — Immunofluorescence microscopy of HeLa cells transfected with a myc-tagged HeV-M expressing plasmid for 12 h (left) or 24 h (right). Nuclei are stained blue, myc-tagged HeV-M green. Sub-cellular localization of HeV-M at 12 h is primarily nuclear, and cytoplasmic at 24 h. (JPG) [file ppat.1005478.s007.jpg]

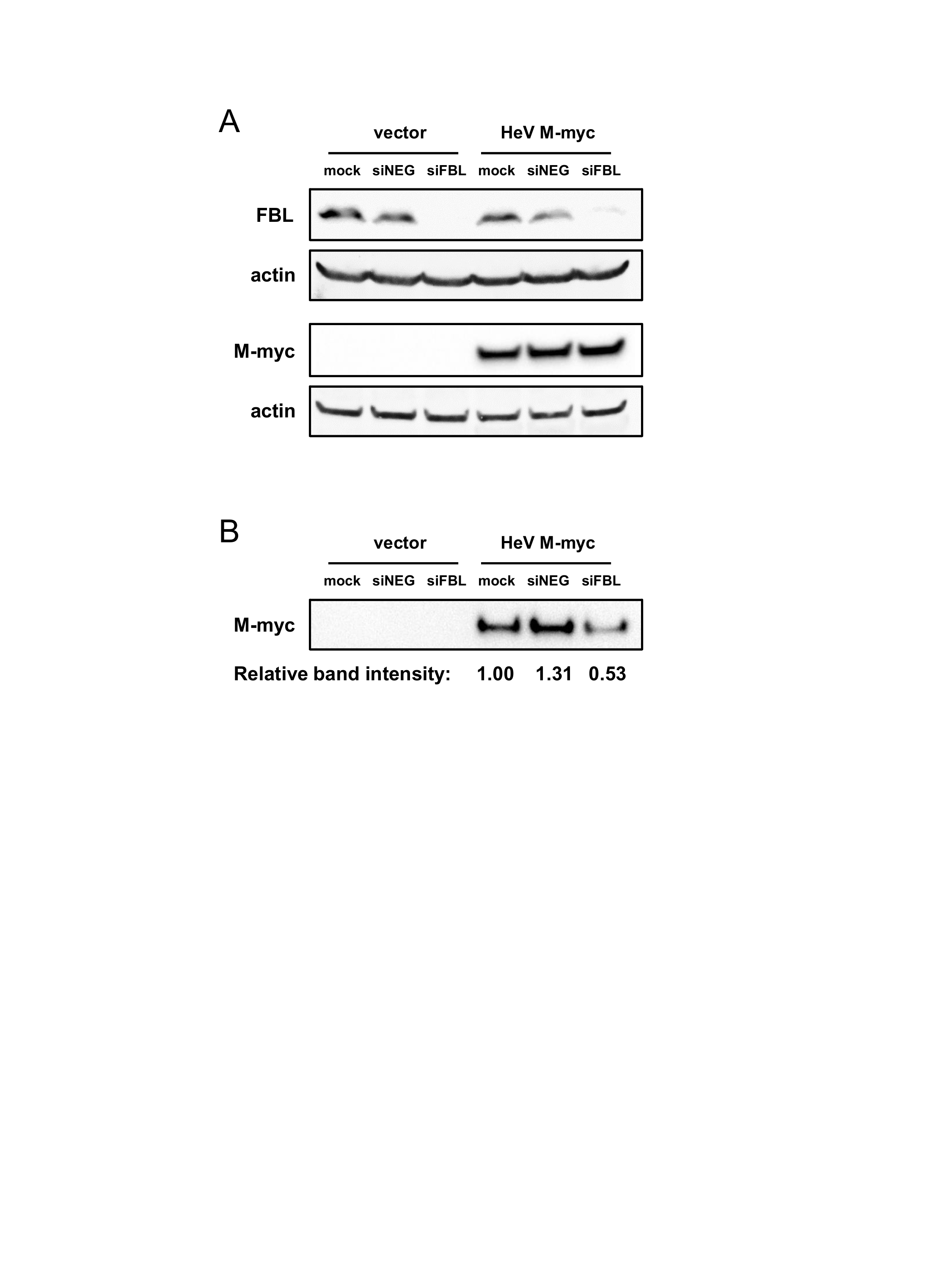

Supplement: S4 Fig — (A) 293T cells undergoing siRNA-mediated knockdown of endogenous FBL were transfected with pCAGGS vector or pCAGGS expressing myc-tagged HeV matrix protein. 48 h post-transfection, whole cell lysates were harvested. Protein expression levels of FBL or M-myc were assessed by Western blotting, using an anti-FBL and an anti-myc antibody, respectively. Detection with an antibody to β-actin were also done as loading controls. (B) Supernatants were harvested from the transfected 293T cells from (A), clarified by low speed centrifugation, and then layered on a 20% sucrose cushion. VLPs were purified by centrifugation at 200,000 ×g for 2 h. VLPs were detected by Western blotting using an anti-myc antibody. Quantification of VLP levels are shown numerically. (TIF) [file ppat.1005478.s008.tif]

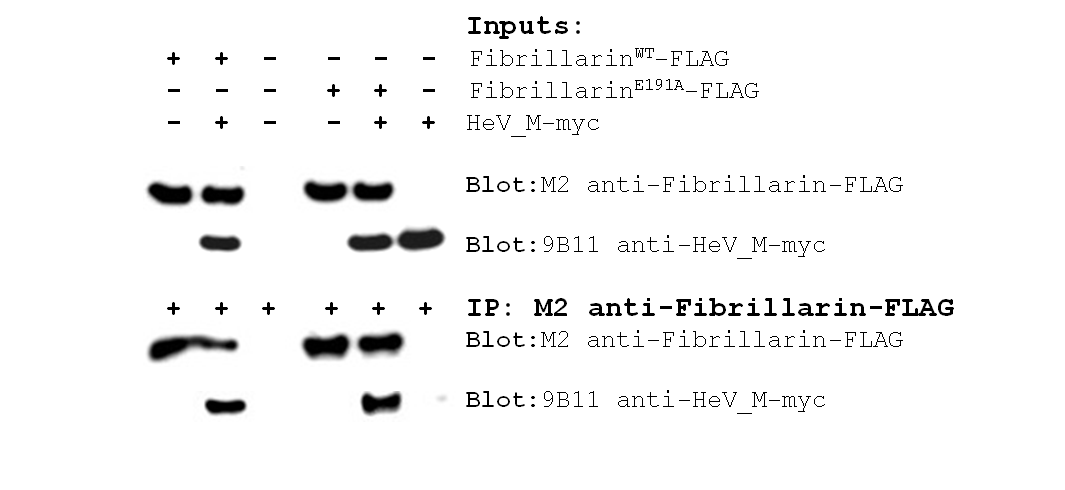

Supplement: S5 Fig — HEK293T cells were transfected to express either FLAG-tagged wild-type or E191A mutant FBL either alone or in combination with myc-tagged HeV-M protein. Lysates were immunoprecipitated with M2 anti-FLAG MAbs, separated by 4%-12% Bis-Tris PAGE and western transferred. Duplicate blots were probed with either M2-HRP or 9B11-HRP to reveal coimmunoprecipitation of HeV-M by FBL. (TIF) [file ppat.1005478.s009.tif]

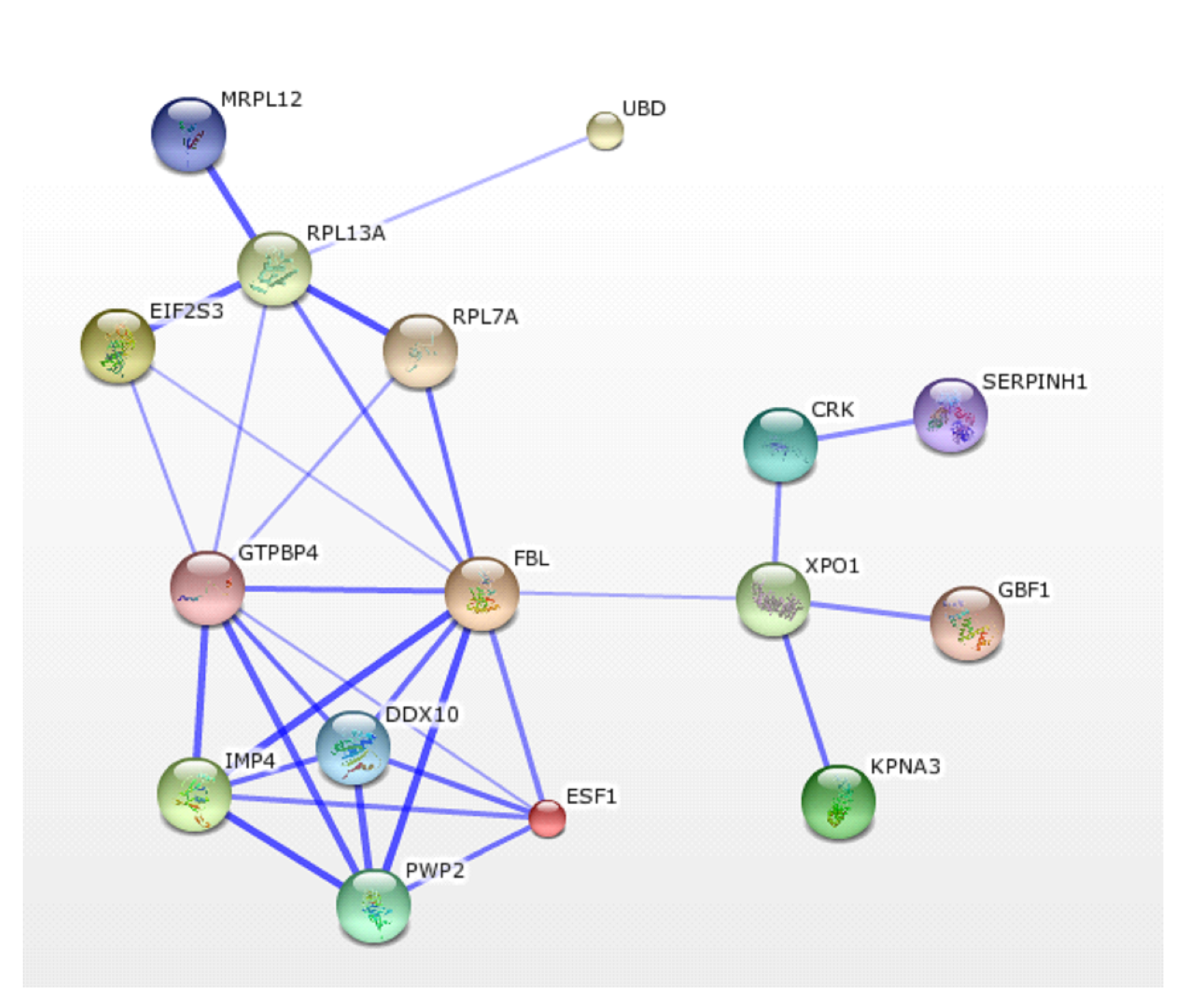

Supplement: S6 Fig — The STRING algorithmic database (Search Tool for the Retrieval of Interacting Genes/Proteins) was used to generate a network view of protein-protein interactions, including direct (physical) as well as indirect (functional) associations (http://string-db.org/) (Szklarczyk et al. 2015). The top 43 candidate hits (as validated by TCID50 virus titration assay; *p<0.05) were used as input data for the database search. Candidates which are not part of the generated network output are not depicted. The thickness of the lines correlate with the confidence scores of the interactions. (TIF) [file ppat.1005478.s010.tif]
